# Bubble rise in molten glasses and silicate melts during heating and cooling cycles

**DOI:** 10.1111/jace.18680

**Published:** 2022-08-15

**Authors:** Lucy E. Jackson, Fabian B. Wadsworth, Joanne Mitchell, Colin Rennie, Edward W. Llewellin, Kai‐Uwe Hess, Donald B. Dingwell

**Affiliations:** ^1^ Department of Earth Sciences Durham University Durham UK; ^2^ Department of Glass and Ceramics The University of Sunderland Sunderland UK; ^3^ Department of Earth and Environmental Science Ludwig‐Maximilians‐Universität Munich Germany

**Keywords:** experiments, fluid dynamics, Stokes, viscous liquid

## Abstract

The Hadamard–Rybczynski equation describes the steady‐state buoyant rise velocity of an unconfined spherical bubble in a viscous liquid. This solution has been experimentally validated for the case where the liquid viscosity is held constant. Here, we extend this result for non‐isothermal conditions, by developing a solution for bubble position in which we account for the time‐dependent liquid viscosity, liquid and gas densities, and bubble radius. We validate this solution using experiments in which spherical bubbles are created in a molten silicate liquid by cutting gas cavities into glass sheets, which are stacked, then heated through the glass transition interval. The bubble‐bearing liquid, which has a strongly temperature‐dependent viscosity, is subjected to various heating and cooling programs such that the bubble rise velocity varies through the experiment. We find that our predictions match the final observed position of the bubble measured in blocks of cooled glass to within the experimental uncertainty, even after the application of a complex temperature–time pathway. We explore applications of this solution for industrial, artistic, and natural volcanological applied problems.

## 1 INTRODUCTION

The behavior of bubbles in silicate melts is of central importance in glass manufacture,^1^, ^2^, ^3^, ^4^, ^5^, ^6^, ^7^, ^8^, ^9^, ^10^ in the science of magma and volcanic eruptions,^11^, ^12^ and in glass art applications.^13^, ^14^ For industrial applications in the glass industry, the rise speed of bubbles in silicate melts is crucial for understanding glass refinement processes that are used to remove imperfections such as bubbles.^1^, ^2^, ^3^, ^4^, ^5^, ^6^ For magmatic applications, bubble rise speeds are central to our understanding of the physics of basaltic eruptions.^15^, ^16^ In glass art, it is desirable to be able to either remove bubbles, or exert a level of aesthetic control over bubble positioning, during the making process.

Existing work has focused on the isothermal rise speed of bubbles, validating the Hadamard–Rybczynski solution for bubble rise in viscous liquids.^2^, ^3^, ^4^, ^17^, ^18^ However, in most industrial, artistic, and natural scenarios, the rise of bubbles occurs in an environment in which temperature may change in time and/or space.^13^, ^19^, ^20^

We can divide non‐isothermal conditions into two types: (1) a temperature change that occurs on the scale of the bubble as it moves, such that the properties of the liquid moving around the bubble may be variable from bubble nose to bubble tail; and (2) a temperature change that occurs approximately homogeneously in the liquid everywhere, but that varies with time. The former case has received attention and solutions have been found for bubbles ascending through a non‐uniform liquid.^21^, ^22^ However, the latter is more relevant to kiln‐based processes or industrial melters in which an entire batch of bubble‐bearing glass may be heated or cooled homogeneously through a complex temperature–time pathway, but where spatial temperature gradients are avoided by design. Here, we focus on this latter case, and use kiln‐based experiments to validate our approach of integrating the Hadamard–Rybczynski equation for changing fluid and bubble properties.

## 2 THEORETICAL BACKGROUND

The Hadamard–Rybczynski equation^17^, ^18^ gives a general solution for the steady‐state velocity $u_{\infty }$ for a bubble of radius *R* moving in a viscous liquid of viscosity μ and is

(1) $$u_{\infty }=\frac{2R^{2}g\left(\rho_{b}-\rho \right)}{3\mu }\beta ,$$

where *g* is gravitational acceleration, ρ_b_ is the density of the bubble, ρ is the density of the liquid, and β=(1+κ)/(2+3κ). Here $\kappa =\mu_{b}/\mu$ with μ_b_ the viscosity of the bubble phase. In the case of a gas bubble μ_b_ is effectively zero, so that κ=0, and β=1/2. Similarly, for a solid particle with $\mu_{b}=\infty$, we see that κ=∞, and β=1/3. In this case, we recover Stokes’ solution for a solid particle in a fluid. Those cases are, respectively, Equations (2a) and (2b),

(2a) $$\;\mathrm{bubble}:\;u_{\infty }=\frac{R^{2}g\left(\rho_{b}-\rho \right)}{3\mu },\;$$

(2b) $$\mathrm{solid}\;\mathrm{particle}:\;u_{\infty }=\frac{2R^{2}g\left(\rho_{b}-\rho \right)}{9\mu }.$$

Equation (1) is derived for the case where the Reynolds number Re is low, such that inertial effects are negligible. In this regime, transients can be neglected, so that $u\to u_{\infty }$ rapidly with respect to other changes of relevance here.^23^ Here, we also solely consider the case where interfacial tension acts to ensure the bubble remains spherical.

In the case of spatially homogeneous temperature change, which we consider here, the timescale for heat conduction must be short compared with the timescale for bubble ascent. The characteristic timescale of conduction in the glass on the bubble scale is $\lambda_{T}=R^{2}/D_{T}$, where *D*_T_ is the thermal diffusivity of the liquid. Similarly, the characteristic steady‐state rise timescale for the bubble is $\lambda_{b}=R/u_{\infty }$. The ratio of these timescales is a thermal form of the Peclet number, Pe_T_

(3) $$Pe_{T}=\frac{\lambda_{T}}{\lambda_{b}}=\frac{u_{\infty }R}{D_{T}}$$

When Pe_T_ ≪ 1, spatial gradients in the liquid temperature decay rapidly compared with the bubble rise process, whereas when Pe_T_ ≫ 1, spatial gradients may influence the bubble rise on the bubble scale. Previous work has investigated the Pe_T_ ≫ 1 case and accounted for the effects of spatial gradients of viscosity and interfacial tension on the bubble scale.^21^, ^22^ Here we focus on the case where Pe_T_ ≪ 1, which is relevant for most laboratory, industrial, and artistic cases (discussed later, in Section 6).

Assuming Pe_T_ ≪ 1, we can numerically integrate Equation (1) or (2) with time, accounting for changes in temperature‐dependent parameters with time. To do this, we must define a temperature–time pathway T(t), which can be some arbitrary functional form. The integral is then

(4) $$x=\int_{t_{0}}^{t}u_{\infty }\left(T\right)dt,$$

wherex is the distance that the bubble rises over the interval between *t*_0_, which is the initial time when the bubble rise starts and some later time *t*, and *T* is some function of time that is known a priori (e.g., a temperature program set by a user in a kiln or furnace). Using Equation (2a) for $u_{\infty }$, we see that the temperature‐dependent parameters may include R(T), $\rho_{b}\left(T\right)$, ρ(T), and μ(T), such that

(5) $$x=\frac{g}{3}\int_{t_{0}}^{t}A\left(T\right)dt$$

where $A=R^{2}\Delta \rho /\mu$, with $\Delta \rho =\rho_{b}-\rho$. In Section 3, we will explore the extent to which the temperature dependence of *R*, ρ_b_, ρ, and μ influences bubble rise.

## 3 EXPERIMENTAL MATERIALS AND METHODS

### 3.1 Experimental materials

The gas–glass system chosen for the experiments is air bubbles in a Spectrum System‐96 glass. This readily available soda‐lime‐silicate glass, near identical to Cristalica glass (in terms of physical properties^24^ the composition of which is reported in Table 1, is used widely in kiln‐based glass art because its working temperature range (776–1126 K), is suitable for many commercial kilns. The relatively high upper working temperature also means it can be manipulated easily without devitrification. These features are key for our experimental methodology. Using air as the bubble phase has two chief benefits: first, it simplifies the experimental process because it is much easier to create a cavity filled with air than a different gas; and second, Spectrum System‐96 glass is saturated with the components of air, making it unreactive with the bubble phase.

**TABLE 1:** Bulk composition of Cristalica glass, a soda‐lime‐silica glass very similar to Spectrum System‐96 glass. Data taken from the manufacturer datasheet

**Oxide component**	**Cristalica glass (wt%)**
SiO_2_	69.0–71.5
TiO_2_	—
Al_2_O_3_	1.1–1.5
FeO	—
MnO	—
MgO	—
CaO	4.0–4.5
Na_2_O	12.5–12.9
K_2_O	5.0–5.5
BaO	2.5–3.0
B_2_O_3_	1.0–1.5
ZnO	0.6–1.3
Sb_2_O_3_	0.2–0.5
H_2_O	n.d.
**Totals**	**95.9–102.2**

The viscosity of silicate liquids can vary over many orders of magnitude across the range of temperatures typical of both volcanic processes and artistic practice.^25^, ^26^, ^27^ For this reason, we deploy a number of techniques to constrain the temperature dependence of viscosity across a range larger than that of the experiments, enabling us to capture its behavior both close to glass transition, and well above it.

**FIGURE 1:**
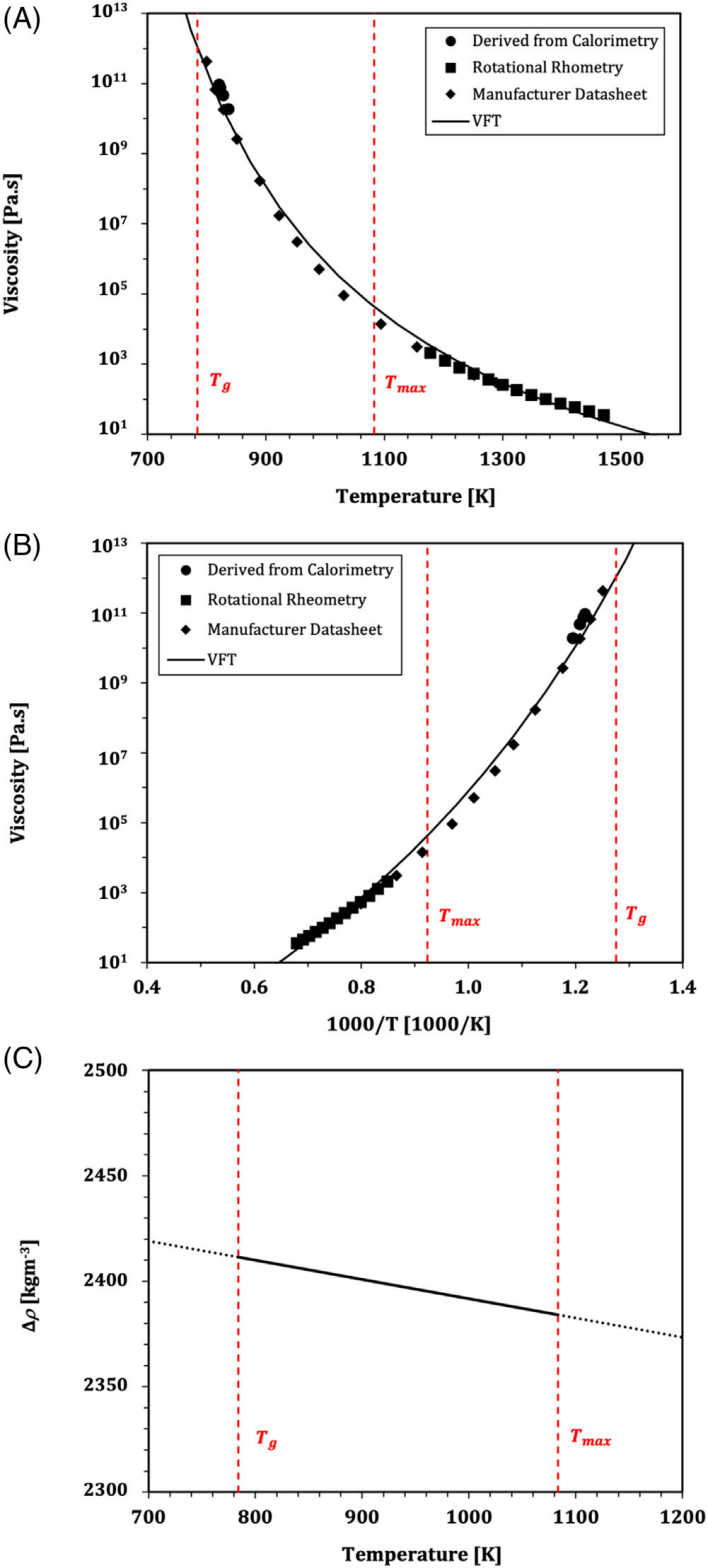
(A,B) The temperature dependence of viscosity for the Spectrum System‐96 glass used in this study. Here we plot viscosity data derived from calorimetry, along with rotational rheometry data and data from the glass manufacturer. The best‐fit to the VFT viscosity model uses *A* = –4.10, *B* = 5700, and *C* = 430. Shown here are the viscosity data and model as a function of (A) temperature *T*, and (B) inverse temperature 1000/T. (C) The temperature dependence of density contrast Δρ between the Spectrum System‐96 glass and dry air contained within the bubble cavity. Boundaries of the experimental temperature range ($T_{g}$‐$T_{max}$) are denoted on all panels by the dashed red lines

First, we measure the relaxation of the glass via differential scanning calorimetry. We place small chips of the glass in a lidded platinum cup in a Netzsch Pegasus 404c Simultaneous Thermal Analysis tool, and heat at different known rates of heating—termed *q*—up to around 900 K. We covered 0.1<q<0.5 K s^−1^. The software associated with the Netzsch instrument was used to find the peak of the glass transition temperature window associated with glass relaxation. Then we used semi‐empirical models for the relationship between peak relaxation temperature and viscosity to constrain the viscosity at the glass transition. Gottsmann et al.^28^ shows that the viscosity at the glass transition temperature $\mu {|}_{T_{g}}$ and the heating rate at which the glass transition temperature is determined are related via

(6) $$\mu {|}_{T_{g}}=\frac{c}{\left|q\right|},$$

where the constant *c* [Pa K] is a function of the glass composition. Gottsmann et al.^28^ provide an empirical model for relating *c* to the composition, showing that *c* is controlled dominantly by the weight percentage of cations in the melt that are excess to the charge balancing roles dictated by the network forming cations. The Gottsmann et al.^28^ empirical model for predicting *c* results in $c\;=\;6.17\;\times 10^{9}$ Pa K for Spectrum System‐96. Equation (1) therefore yields values for μ at $T=T_{g}$ (Figure 1), which moves with heating and cooling rate.

Second, we use a rotational rheometer in which crushed chunks of the glass are loaded into large platinum crucibles and held at 1300°C for 12 h, ensuring homogenization to a single‐phase liquid. A platinum‐coated spindle^29^ is lowered into the melt and controlled using a Brookfield HBTD which can apply rotation speeds of 0.5–50 rpm. The apparatus, technique, and data processing are described by Dingwell.^30^ The technique involves a series of temperature reduction steps, rotating the spindle until the measured torque equilibrates at each temperature before moving to the next. The equilibrium torque is then proportional to the shear stress, which, together with the rotation rate, can be used to compute the shear viscosity. Finally, we check the direct measurements reported here against the data for μ(T) provided from the manufacturer. These data are shown together in Figure 1 and are given in Table 2.

**TABLE 2:** Viscosity data for Spectrum System‐96 derived from calorimetry and rotational rheometry. μ(T) data provided by the glass manufacturer is also listed. These data are plotted in Figure 1 to show the temperature dependence of the glass viscosity and estimate a VFT fit

**Rotational rheometry**		
**Temperature (°C)**	**Temperature (K)**	**log| Viscosity | log| *Pa s* |**
1198	1471.15	1.545
1173	1446.15	1.644
1149	1422.15	1.756
1125	1398.15	1.870
1100	1373.15	1.990
1076	1349.15	2.117
1051	1324.15	2.252
1027	1300.15	2.403
1003	1276.15	2.554
978	1251.15	2.722
954	1227.15	2.898
930	1203.15	3.093
905	1178.15	3.310

**Calorimetry**		
**Temperature (°C)**	**Temperature (K)**	**log| Viscosity | log| *Pa s* |**
554.98	828.13	10.67
563.09	836.24	10.27
554.48	827.63	10.67
548.01	821.16	10.97
563.79	836.94	10.27
550	823.15	10.87

**Data from manufacturer**		
**Temperature (°C)**	**Temperature (K)**	**log| Viscosity | log| *Pa s* |**
526.67	799.82	11.64
541.21	814.36	10.83
554.55	827.70	10.26
577.58	850.73	9.42
616.36	889.51	8.22
649.09	922.24	7.23
679.39	952.54	6.48
716.97	990.12	5.70
758.18	1031.33	4.96
821.21	1094.36	4.15
881.82	1154.97	3.49
978.79	1251.94	2.68
1015.15	1288.30	2.47

Using both the calorimetric data, the rotational rheometry data, and data provided by the manufacturer, we arrive at constraint of μ(T). The temperature dependence of the glass viscosity μ(T) is well described for viscous fluids such as molten glass through the Vogel–Fulcher–Tammann (VFT) equation

(7) $$\log\left|\mu \right|=A+\frac{B}{T-C},$$

where *T* is the temperature of the glass (here in Kelvin) and *A*, *B*, and *C* are constants specific to the glass. By minimizing the sum of square residuals between our data and the predictions made by Equation (7), we find a best fit between our data and Equation (7) using *A* = −4.10, *B* = 5700, and *C* = 430. We note here that when soda‐lime‐silica glass is held at 1300°C for 12 h it is possible that volatilization of light elements such as sodium may occur, affecting the melt viscosity. However, given our measured results for viscosity have a near identical match to results from the manufacturer datasheet, we conclude it is not a factor to take into consideration here.

Both the gas density and the glass density vary far less substantially than the glass viscosity, and so could, in principle, be taken to be constant. However, for completeness we include their temperature dependence here and discuss later the effect of neglecting or accounting for these effects. The ideal gas law gives a form for the temperature‐dependence of gas density as $\rho_{b}=Pm_{g}/\left(RT\right)$, where *m*_g_ is the molecular weight of the gas, *S* is the gas constant and Pand *T* are the pressure and temperature of the system, respectively. Taking $P=10^{5}\;$Pa, $m_{g}=0.029\;$kg mol^−1^ for dry air, R=8.31J K^−1^ mol^−1^, we find that over the range $T_{g}$‐$T_{max}$ (784.15–1083.15 K), ρ_b_ varies from $\rho_{b}=1.22\;$kg m3 to $\rho_{b}=0.35\;$kg m3. For the density of the glass phase ρ, we use $\rho =\rho_{0}+\alpha T$, where $\rho_{0}=2483.8$ kg m3 is the extrapolated zero‐temperature value of ρ, and α=−0.09183K^−1^ represents the temperature‐dependence of the density. These coefficients are found by inputting a glass composition from a manufacturer datasheet (Table 2) into a glass density model calculation^31^ which outputs a linear relationship of the form given for ρ(T). This results in an expected ρ=2408.2 kg m3 at the glass transition, down to ρ=2367 kg m3 at 1270 K. In Figure 1C we give the value of $\Delta \rho \;=\;(\rho -\rho_{b})$ over the temperature range of interest.

Bubble radius also varies as a function of temperature, in response to expansion and contraction of the gas phase. Above *T*_g_, we integrate the bubble radius in Equation (5) by assuming Charles's Law holds at each temperature so that $R\left(t\right)=R_{0}\sqrt[{3}]{T\left(t\right)/T_{g}}$.

### 3.2 Experimental method: Adaptation of an artist's method

To experimentally validate the analytical model for bubble rise presented in this study, we adopt a method used by Mitchell^13^ for artistic purposes, and which is described here. Thin glass sheets (provided by the manufacturer) are layered. One sheet contains a precision waterjet‐cut cylindrical hole. This cut sheet is placed one sheet from the bottom such that there are glass sheets above this cut sheet, and at least one glass sheet below. The pile of glass sheets is then loaded in a kiln and supported on the sides by refractory kiln shelf material for support and to prevent slumping on heating.

**FIGURE 2:**
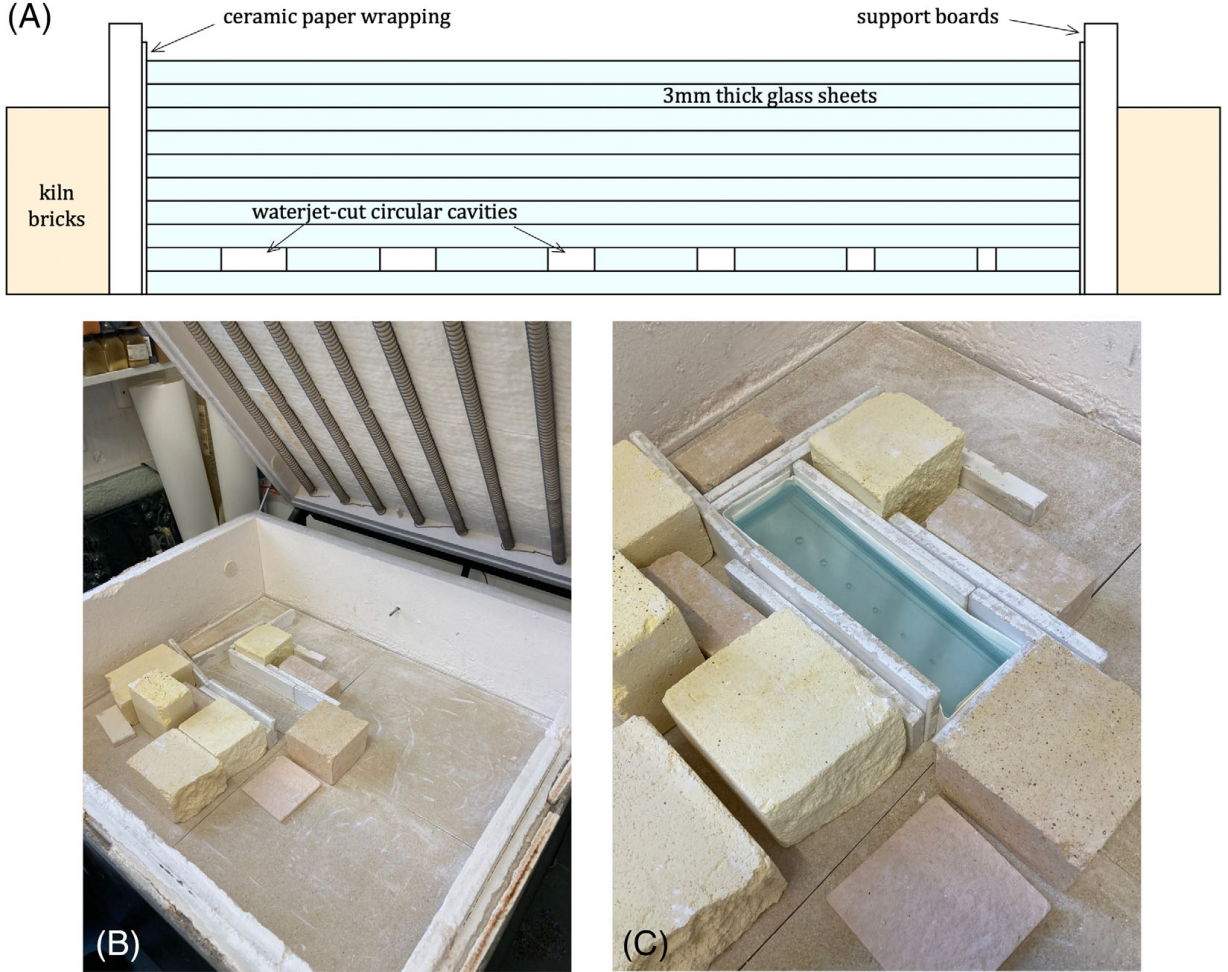
Images outlining the glass and kiln setup used in the experimental procedure. (A) Schematic diagram showing how sheets of Spectrum System‐96 soda‐lime‐silicate glass are stacked together into a block, with one sheet containing several circular waterjet‐cut cavities. The wrapping and supports used to prevent the glass from slumping when molten are also shown. (B) Photograph of the kiln box with some of the support boards and kiln bricks in place. The heating elements can be seen on the lid of the kiln box, and the thermocouple used to read the kiln temperature can be seen positioned in the center back wall of the kiln. A secondary thermocouple to compare target and observed temperatures was placed closer to the glass position. (C) Photograph showing how the glass stacks were positioned prior to heating. This image shows block *Ic‐H1*, which is 30 cm in length and 10 cm wide

**FIGURE 3:**
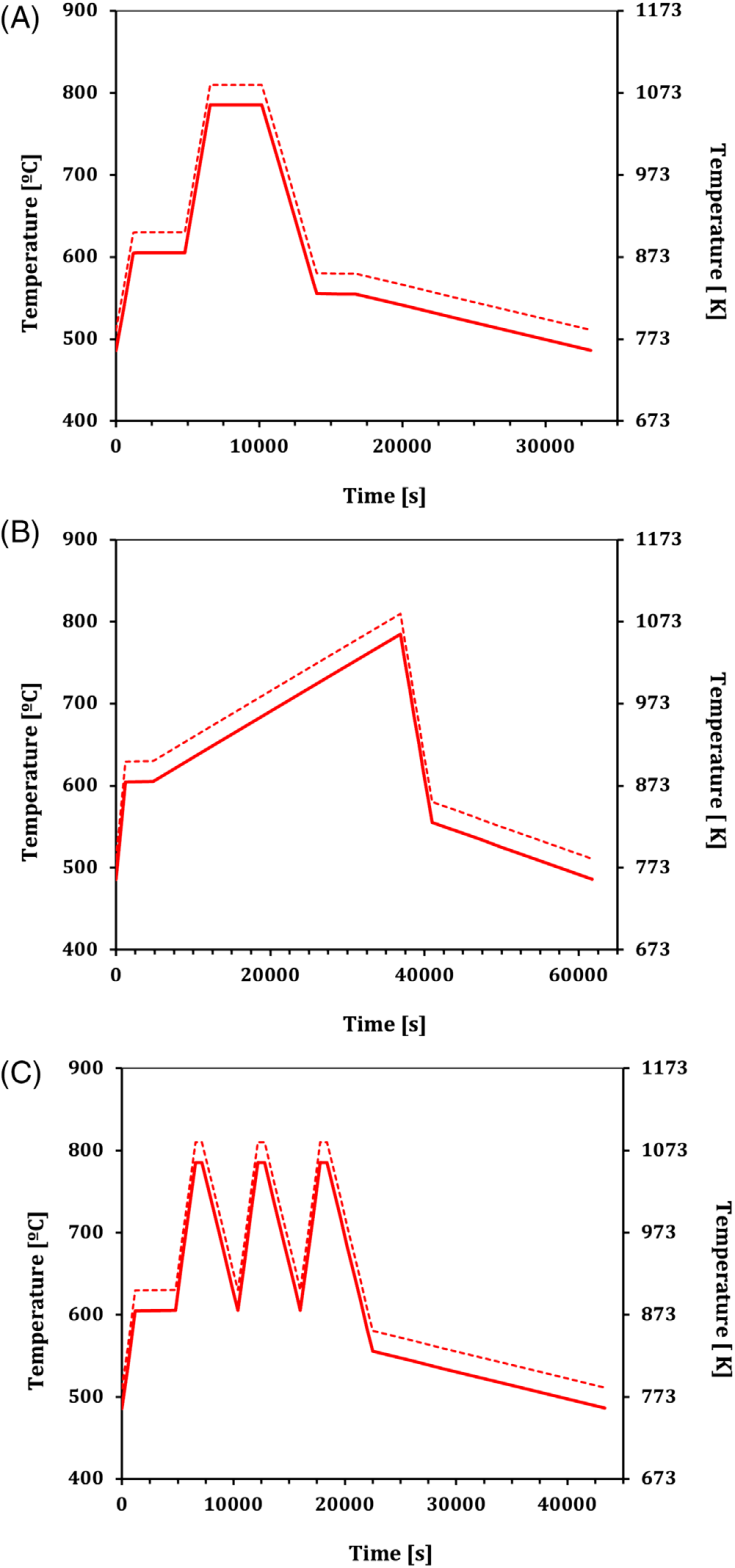
The three different T(t) programs used in the glass kilns to test Equation (5). In each case t=0 is taken to be the time at which $T=T_{g}$, which is the temperature at which the glass relaxes to a molten state. The dashed red line shows the kiln temperature programmed before the experiment, and the solid red line shows the true kiln temperature, accounting for the −25 K offset observed via thermocouple readings. (A) Program 1 with a single isothermal hold. (B) Program 2 with a slow heating ramp then rapid cooling to annealing temperature. (C) Program 3 with three successive cycles of heating and cooling before annealing

**FIGURE 4:**
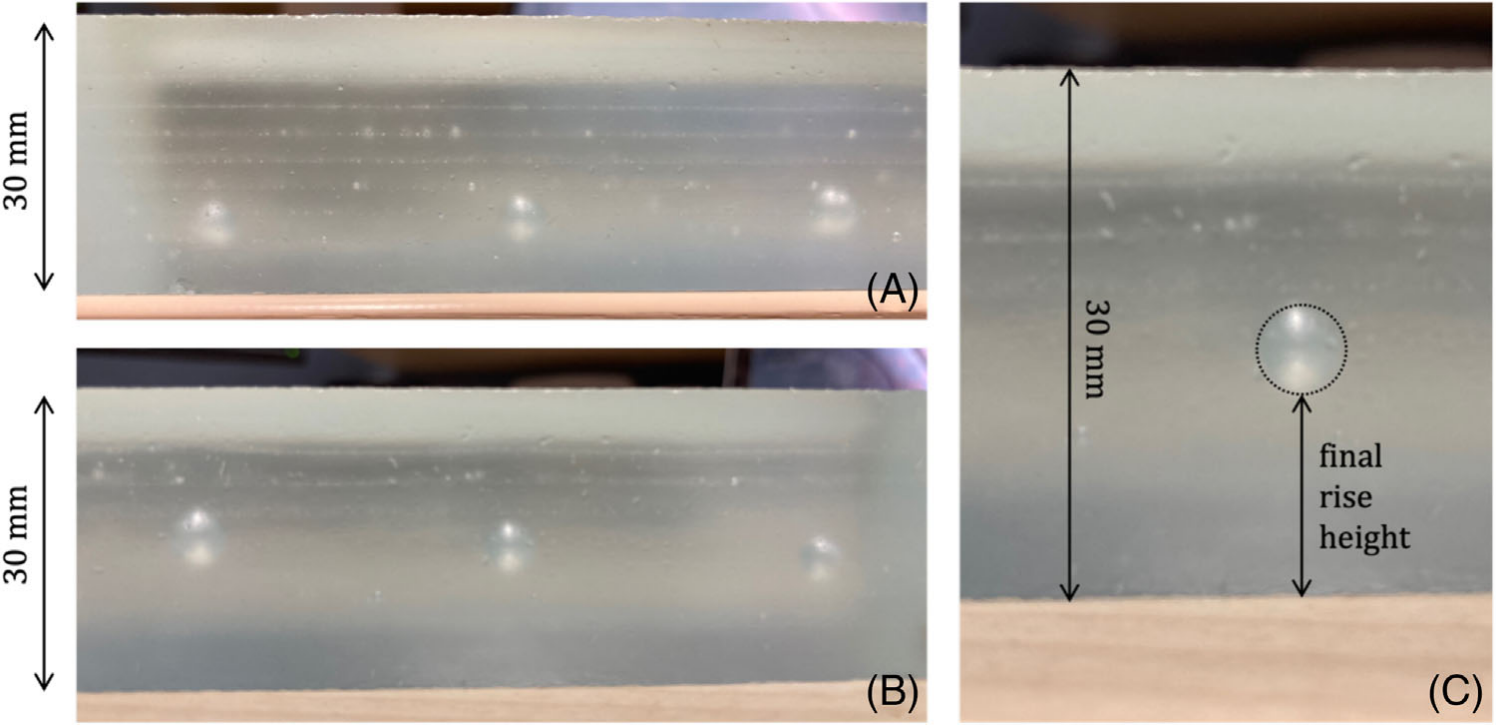
Images showing bubbles trapped within the fused glass blocks, post‐heating. (A) Bubbles within block *Ic‐H2* measuring, 2.5, 3.0, and 3.5 mm in radius from left to right. (B) Bubbles within block *Ic‐H1* measuring, 2.5, 3.0, and 3.5 mm in radius from right to left. (C) Diagram showing how digital measurements of final bubble position are made. Distance from the base of the block to the base of the bubble is measured and scaled from a digital measurement of block height, which is known in each case. For *Ic‐H1*, the block height is 30 mm. This method was completed for all bubbles in all five blocks

Heating is achieved by setting kiln programs with defined T(t) profiles (Figure 3). When the glass is heated above a temperature at which the glass relaxes (taken here to onset around the glass transition temperature *T*_g_), the sheets fuse into a single block over a relatively short surface–surface healing time^32^ and allow the bubbles trapped inside to relax to be spherical and rise buoyantly. In Figure 2, we illustrate aspects of this methodology. Applying this method, we produced starting samples with cylindrical cavities ranging in size from 1.0 to 3.5 mm radius precision‐cut into a sheet of the Spectrum System‐96 glass. The cavities are sufficiently far apart such that the bubbles will not coalesce or interact during rise. Following the application of the kiln program, including annealing, the fused block is removed, cut, polished, and the bubble positions measured (Figure 4).

In order to apply Equation (5) to analyze bubble rise, we define *t*_0_ as the time in the T(t) program at which$\;T\;=\;T_{g}$. We take this to be the temperature at which $\mu =10^{12}$ Pa s (IUPAC standard) calculated using Equation (7). This assumes that the equivalent spherical radius of the cavity cut through the glass sheet, *R*_d_, is equal to the radius of the bubble at *t*_0_ (or $T=T_{g}$); this is justified because the glass sheets remain unfused below *T*_g_ and therefore gas can escape from between the stacked sheets as gas expands between room temperature and the glass transition. Considering this, we apply three different T(t) programs to test Equation (5).

1. Heating at 0.1 K s^−1^ to 1083 K, an isothermal hold for 3600 s, followed by initial cooling at 0.06 K s^−1^, to 853 K, at which the sample was held for 2700 s, then a slower annealing cool at $4.2\;\times 10^{-3}$ K s^−1^, down to *T*_g_, then room temperature (Figure 3A).
2. Heating at $5.6\;\times 10^{-3}$ K s^−1^ from 878 to 1083 K, no isothermal hold, followed by initial cooling at 0.056 K s^−1^, to 853 K, then slower cooling at $3.3\;\times 10^{-3}$ K s^−1^, down to *T*_g_, then room temperature (Figure 3B).
3. Three repeated cycles of heating and cooling at 0.1 and 0.056 K s^−1^, respectively, between 878 and 1083 K, ending with a cool to 853 K and a slower annealing cool at $4.2\;\times 10^{-3}$ K s^−1^, down to *T*_g_, then room temperature (Figure 3C).

These different kiln cycles vary the viscosity of the glass at different rates, which will in turn control the terminal rise velocities of the bubbles and therefore their final position within the glass block (Equation 5). Kilns often have an offset between target temperatures and actual observed temperatures. To account for this, we included a thermocouple in the kiln chamber, and recorded the temperature of the kiln atmosphere within a few centimeters of the samples. The measured thermocouple reading remained consistent at multiple positions and times during any of the heating cycles, leading us to conclude that temperature was uniform throughout, despite the size of the kiln.

On average, this resulted in a −25 K offset between the set temperature and the measured temperature, which is applied hereafter to our results.

### 3.3 Measuring bubble rise

Five different experimental runs were completed, using glass sheet stacks of varying size, each containing five or six bubbles of differing radii. These runs cover all three of the different kiln programs for T(t). Following removal from the kiln, the sheets of glass layers are fused into a single coherent block. In each case, the bubbles are enclosed in the glass and have visibly risen from the initial position. These final rise heights $h_{f}$ are determined digitally from scaled photographs of each block (Figure 4), to determine the total distance moved by each bubble. To do this, the images were scaled to the height of the block which remained constant throughout, and *h*_f_ measured as the distance from the block base to the lower interface of the bubble in its final position.

To compute the distance travelled by each bubble over the duration of each T(t) kiln programs, initial height *h*_0_ is taken as the distance between the block base to the base of the cavity‐containing sheet. The uniform manufacture of the glass sheets used to make up the block means *h*_0_ is easily calculable with negligible uncertainty.

## 4 RESULTS AND ANALYSIS

Visual observations of the fused glass block show that bubbles with a larger initial radius had risen further than those with a smaller radius for the same kiln program. There is also a visible difference in the position of bubbles with the same radius that were subjected to the different kiln programs.

In Table 3, we present bubble rise data from all of the experimental runs, giving the initial height *h*_0_, final height *h*_f_, apparent total rise height $h_{f}-h_{0}$, and uncertainty for each case. The rise heights of three bubbles are removed from the dataset here as the top of the glass block was visibly deformed and thought to have influenced the movement.

**TABLE 3:** Bubble rise data from all five experimental blocks, covering the three different kiln programs tested. The three rows of indicated with an asterisk are removed from further analysis as the movement of these bubbles is thought to have been influenced by their close proximity to the top of the block

Glass block	Kiln program	Initial bubble radius (*R*) (mm)	Initial height (*h*_0_) (mm)	Final height (*h*_f_) (mm)	Apparent total rise height (mm)	Uncertainty ± mm
Cl‐Lg	1	1.0	6.0	6.45	0.45	0.10
Cl‐Lg	1	1.5	6.0	7.35	1.35	0.10
Cl‐Lg	1	2.0	6.0	8.37	2.37	0.10
Cl‐Lg	1	2.5	6.0	9.50	3.50	0.11
Cl‐Lg	1	3.0	6.0	10.40	4.40	0.10
Cl‐Lg	1	3.5	6.0	11.41	5.41	0.10
Cl‐Sm	1	1.0	6.0	6.50	0.50	0.14
Cl‐Sm	1	1.5	6.0	6.59	0.59	0.14
Cl‐Sm	1	2.0	6.0	7.68	1.68	0.13
*Cl‐Sm	1	2.5	6.0	8.17	2.17	0.14
*Cl‐Sm	1	3.0	6.0	8.50	2.50	0.14
*Cl‐Sm	1	3.5	6.0	9.12	3.12	0.15
Ic‐Sm	1	1.5	6.0	7.40	1.40	0.15
Ic‐Sm	1	2.0	6.0	8.32	2.32	0.15
Ic‐Sm	1	2.5	6.0	9.69	3.69	0.15
Ic‐Sm	1	3.0	6.0	10.05	4.05	0.15
Ic‐Sm	1	3.5	6.0	11.94	5.94	0.15
Ic‐H1	2	1.0	9.0	9.89	0.89	0.10
Ic‐H1	2	1.5	9.0	11.08	2.08	0.10
Ic‐H1	2	2.0	9.0	12.94	3.94	0.10
Ic‐H1	2	2.5	9.0	14.57	5.57	0.10
Ic‐H1	2	3.0	9.0	16.65	7.65	0.10
Ic‐H1	2	3.5	9.0	18.28	9.28	0.10
Ic‐H2	3	1.0	6.0	8.16	2.16	0.11
Ic‐H2	3	1.5	6.0	8.65	2.65	0.11
Ic‐H2	3	2.0	6.0	9.14	3.14	0.11
Ic‐H2	3	2.5	6.0	10.18	4.18	0.11
Ic‐H2	3	3.0	6.0	10.80	4.80	0.11
Ic‐H2	3	3.5	6.0	11.45	5.45	0.11

Here, we compare the apparent bubble rise distances with two predictions: (1) an isothermal prediction for the average bubble rise velocity, where we make some assumption about a characteristic temperature for each kiln program in order to assign as single bubble velocity via Equation (2); (2) a non‐isothermal prediction for the final bubble height via Equation (5).

### 4.1 Isothermal assumption

It is useful to test if a final bubble position can be adequately determined using an isothermal approximation to predict the bubble velocity. To do so, we use $u\;=\;(h_{f}-h_{0})/\Delta t$ as the experimental measure of the average bubble velocity, where Δtis the time available for bubble rise. We take Δt to be the total time spent at $T>T_{g}$. In order to compare *u* with $u_{\infty }\;$given in Equation (2), we also assign a single characteristic temperature ⟨T⟩ to each kiln program. In the case of the first kiln program, which is a ‘‘standard’’ isothermal hold, we take ⟨T⟩ as being the isothermal hold temperature. For the other more complex kiln programmes, we take ⟨T⟩ to be an average temperature taken as the mean of the whole program above *T*_g_. For each kiln program in turn, the times Δt and temperatures ⟨T⟩ are

1. For T(t) program 1: Δt=33,144s and ⟨T⟩=1053 K.
2. For T(t) program 2: Δt=61,755s and ⟨T⟩=963 K.
3. For T(t) program 3: Δt=43,345s and ⟨T⟩=963 K.

**FIGURE 5:**
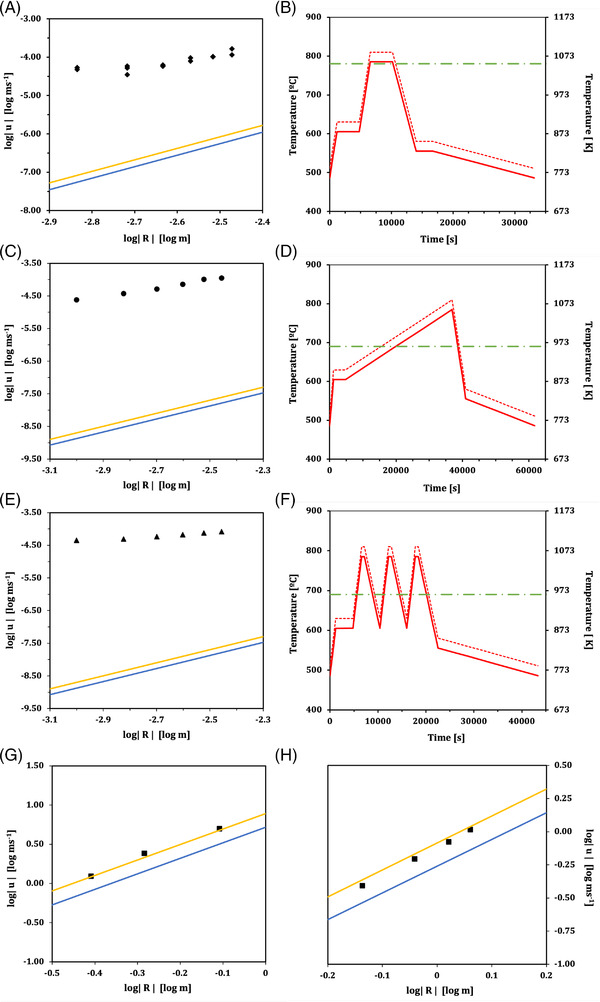
Comparing observed and modelled bubble rise velocities for a range of bubble sizes and temperature programs. The black data points show bubble velocities calculated from experimental observations of bubble rise. The solid blue and yellow lines show the Stokes (Equation 2b) and Hadamard–Rybczynski (Equation 2a) solutions for the velocity of correspondingly sized bubbles, respectively. On the temperature–time graphs, the solid red line shows the kiln program (with −25 K correction) used for that experimental data set, the dashed red line shows the set temperature programmed into the kiln, and the green dot‐dashed line shows the assumed isothermal temperature used in the calculation of the Stokes and Hadamard–Rybczynski solutions. This green dot‐dashed line also accounts for the −25 K temperature offset. (A,B) Kiln program 1 that produced data from blocks *Cl‐Lg, Cl‐Sm*, and *Ic‐Sm*. (C,D) Kiln program 2 used to produce data from block *Ic‐H1*. (E,F) Kiln program 3 used to produce data from block *Ic‐H2*. Across all three graph‐sets, the velocities calculated from experimental data have a poor fit to either of the two modeled solutions. Results for isothermal bubble rise collected from experimental work by (G) Hornyak and Weinberg,^2^ and (H) Li and Schneider,^5^ are also shown for completeness and comparison

To calculate $\mu_{\infty },\;$we find the value of $\rho_{b}$, ρ, and μ, at ⟨T⟩, via the material property calculations introduced in Section 3. Comparing these calculated velocities to the experimentally derived velocities across a range of bubble sizes (Figure 5) shows a poor fit in all cases. The experimental bubble rise data lies closer to the Hadamard–Rybczynski solution than the Stokes solution in each case. This highlights that assuming a single isothermal temperature for the duration of the experiment is an over‐simplification that yields results which do not match well with observations. Furthermore, it justifies the need for an alternative approach for non‐isothermal conditions such as our solution presented here in Equation (5), particularly for complex heat–cool cycles. For completeness, we also show the results for truly isothermal bubble rise experiments performed previously,^2^, ^5^ in order to confirm that in this idealized isothermal case, the Hadamard–Rybczynski solution outperforms the Stokes solution, as expected from the derivation (Figure 5; Equation 2).

### 4.2 Non‐isothermal conditions

Here, we compare the experimental results for bubble rise height to those predicted by a non‐isothermal solution for bubble rise (Equation 5). To do this, we take three different solutions of Equation (5) that account for varying complexity, in order to determine the extent to which integration of Δρ, *R*, and μ affects the accuracy with which we can predict the final bubble position.

First, we note that the viscosity μ varies most substantially over temperature ranges relevant to the kiln programs used here. Therefore, we first define a minimal model in which it is only μ that is integrated in Equation (5), and bubble radius *R* and density contrast Δρ are kept constant. Second, we define a model in which both μ and *R* are integrated, while Δρ is constant. Finally, third, we define a model in which all parameters are integrated with the changing temperature–time program. All of these are given by Equation (5), but represent situations in which different information may be available for a given bubble–glass system, and so are worth testing individually.

In all cases, we take *t*_0_ to be the time when *T*_g_ is met on heating, and then numerically compute the bubble position at a series of given time intervals. At each time the values of the temperature‐dependent parameters are found and used to calculate the change in bubble position during that time interval.

**FIGURE 6:**
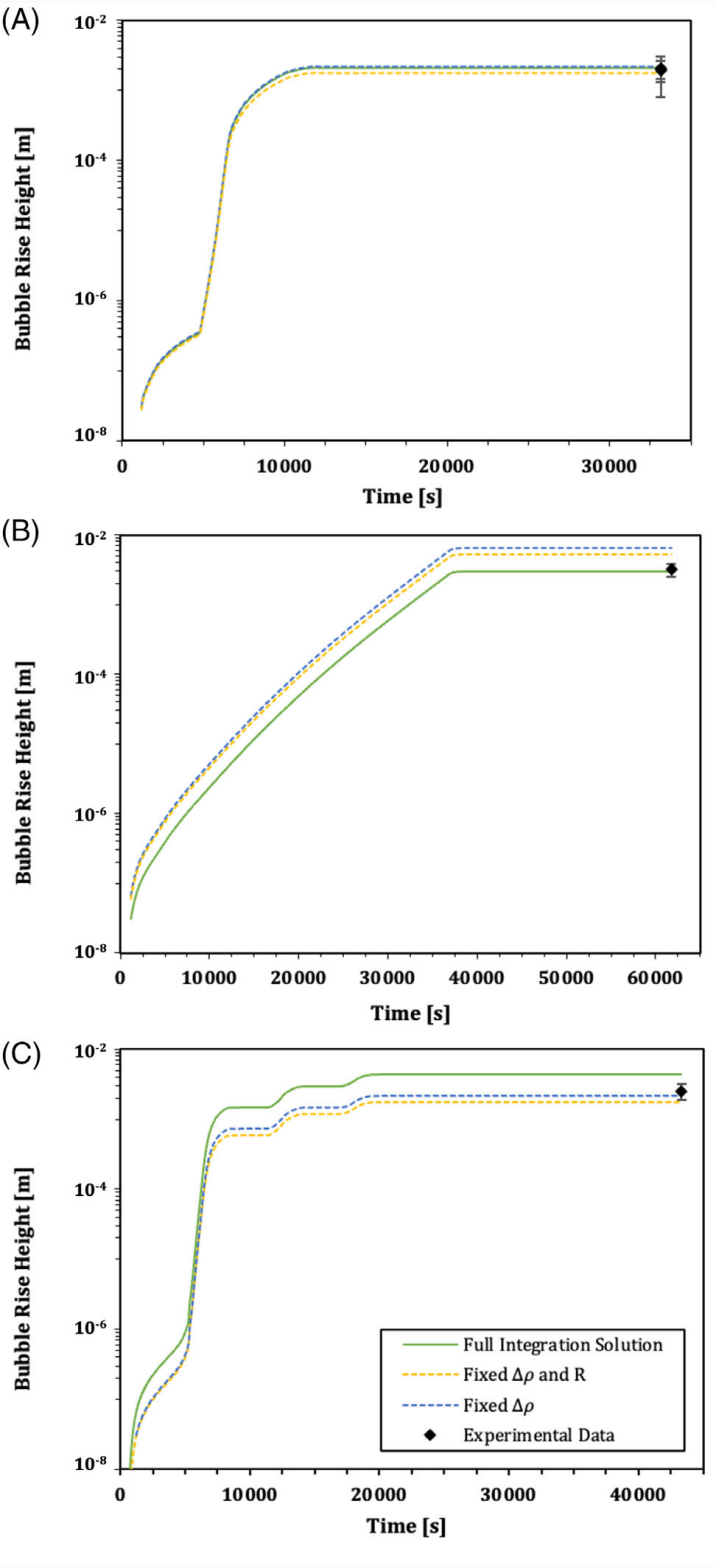
Model outputs showing the motion of a 2.0 mm radius bubble during the time the system was held at a temperature above *T*_g_ via (A) kiln program 1, (B) kiln program 2, and (C) kiln program 3. On each graph, different solutions to Equation (5) are shown. The solid green curve shows the complete solution where R(T), Δρ, and μ(T) are all integrated for. The dashed blue line shows the solution where R(T) and μ(T) are integrated for, and the gas–melt density contrast is fixed. The dashed yellow line shows the solution where only viscosity is integrated with respect to time, μ(T). The final, experimentally observed positions of 2.0 mm radius bubbles in each setting are shown as black data points. Similar output graphs can be produced for different sized bubbles and for the different kiln programs

**FIGURE 7:**
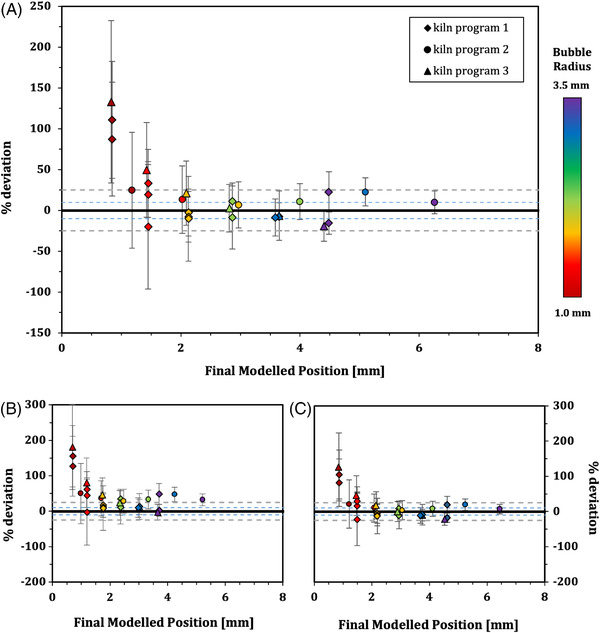
Percentage deviation of observed final bubble rise heights from those modelled by the various solutions to Equation (5): (A) the full integration solution, (B) ‘‘fixed Δρ and *R*’’ solution, and (C) ‘‘fixed Δρ’’ solution. In each case, this percentage deviation is plotted against the final modeled bubble position. Each data point is colored according to the initial bubble radius, and the shape of the point indicates the kiln program. Uncertainty in the observed final position is used to generate the vertical error bars. The grey dashed lines show a 25% deviation from the model, and the blue dashed lines a 10% deviation from the model. These demonstrate that all complexities of model solution provide a better fit for the motions of larger bubbles than smaller ones, and that the full integration solution provides the best fit overall

In Figure 6 we show an example of the comparison between these three variants of the non‐isothermal model (Equation 5) compared with the observed rise height $h_{f}-h_{0}$ for a bubble of 2 mm initial radius for each of the three different T(t) kiln programs. This analysis shows that all three of these solutions provide a reasonable prediction for the final rise height of the bubble, suggesting that integrating for the time evolution of the viscosity is the most important effect to account for here. A comparison of all observed bubble rise heights with those predicted by Equation 5, integrating for all temperature‐dependent parameters, is shown in Figure 7. Across all bubble sizes > 1 mm and varying complexities of heating and cooling cycles, the measured bubble position is well‐predicted to be within 25% of the observed value. The smallest bubbles are poorly predicted, which is likely to be due to poor resolution on the initial and final heights of these bubbles.

Figure 7 also shows data points for the more simplistic integration solutions to Equation (5) where Δρ,R, or both are kept constant. These also show a reasonable fit to observations, showing that viscosity is the first‐order control on bubble rise in non‐isothermal conditions. This implies that, understanding the viscosity–temperature relationship of a silicate melt is crucial to being able to define bubble rise when temperature is not constant, and that reasonable estimations of bubble position can be made with this information alone.

## 5 DISCUSSION

In this work, we have shown that non‐isothermal effects on bubble motion cannot be ignored, even for relatively simple cooling and heating programs typical of glass forming processes. We provide and validate a simple integral approach to predicting the rise height of bubbles in molten glass, based on the Hadamard–Rybczynski equation (Equation 2a). Here, we discuss potential applications of this to industrial, natural, and artistic situations, before discussing future work.

### 5.1 Applications

Bubble rise in silicate melts is a key process in industrial, natural, and artistic application scenarios. Typically, such bubbles in silicate melts are small and spherical, and therefore the Stokes or Hadamard–Rybczynski solutions for the terminal steady‐state rise velocity are used. However, across those same domains of application, temperature is rarely constant. For example, in natural silicate melts containing bubbles, such as magmas, there are myriad ways that temperature can vary during cooling of lava, or as a natural feature of magma rising in the Earth's crust.^33^ In artistic settings, glass containing gas elements may be subject to tailored kiln programs to help control rise,^13^, ^34^ and bubbles in vats of glass for glassblowing may rise toward a free surface that is at a lower temperature. In industrial settings, glass containing unwanted bubbles may be flash‐heated to remove the bubbles during bubble refinement processes.^1^, ^6^ For all cases, we propose that our integral solution for bubble displacement is of wide utility.

#### 5.1.1 Volcanology

In magmatic silicate melts, it is important to know the conditions under which bubbles are coupled to or decoupled from magma that is rising up through the crust.^35^ In this scenario, the natural comparison is to assess the ratio $u_{\infty }/u_{m}$, where *u*_m_ is the average magma ascent velocity and where $u_{\infty }/u_{m}\gg 1$ indicates decoupled bubbles that rise through the magma. This ratio is a Stokes number, and requires explicit knowledge of $u_{\infty }$. In the case of convective overturn in open volcanic vents, such as lava lakes, there can be a substantial temperature difference between the magma at depth and the magma at the surface,^36^ such that isothermal assumptions for computing $u_{\infty }$ may be inappropriate. Our Equation (5) can be mapped onto a known temperature field in order to assess the extent to which bubbles are coupled during convective overturn. Similarly, as lavas cool, the bubbles within them can rise a certain distance, leading to characteristic bubbly layering.^37^ This question can be addressed by mapping $u_{\infty }$ to a known cooling trajectory for the lava T(t). Magmatic systems are vertically extensive, such that rising bubbles not only experience changes in temperature, but also experience very large changes in pressure. As a result, their radius may evolve via the ideal gas law, and not only via changes in temperature captured by the simplified Charles’ law. This can be incorporated into our Equation (5) by redefining R(t) in *A*.

#### 5.1.2 Artistic methods

**FIGURE 8:**
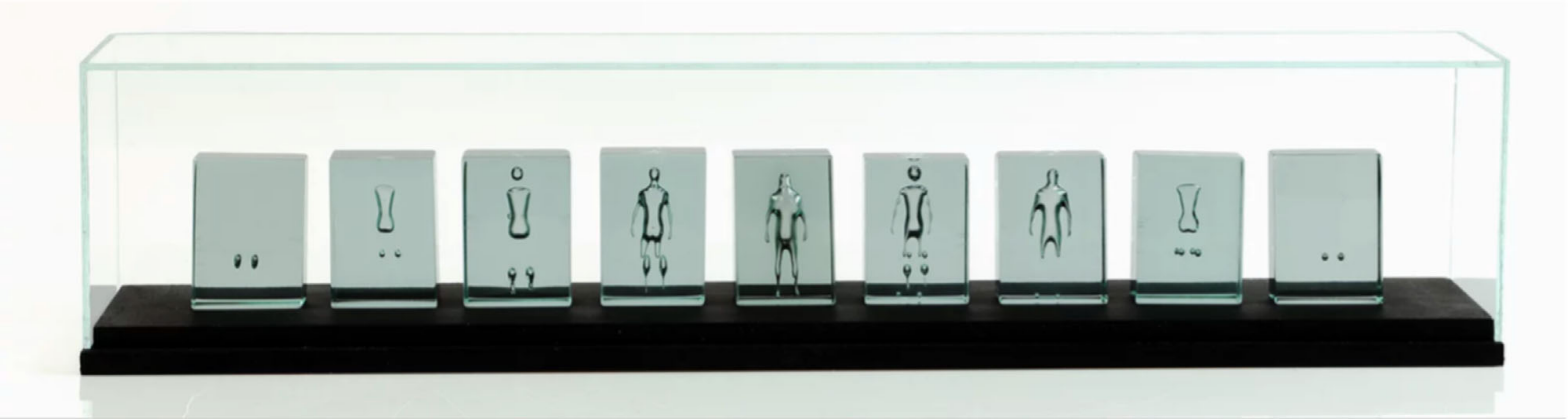
Image of glass artwork ‘‘Deconstructed Being’’ by Joanne Mitchell. Each of the nine blocks were created through bubble entrapment and the use of kiln‐controlled heating of soda‐lime‐silica glass with the specific requirement that the bubble rise velocity is kept low. Dimensions: 80 × 15 × 15 cm. Photography by Colin Rennie

The use of kiln‐controlled heating and cooling programs in the creation of glass art gives a specific application of this model. Ariel or precision air entrapment are methods which rely on the ability of the artist to control the shape or migration of air bubbles in glass through controlled heating processes (Figure 8). To achieve the desired artistic effect requires an understanding of the material behavior so that the heating and cooling cycles of a kiln, or the soak period (isothermal hold) of the glass piece can be adapted accordingly. The precision air‐entrapment method that was adapted for the experimental validation in this study is used by glass artists to create intricate gas‐bearing glass artworks in which the artist seeks to control bubble rise within particular aesthetic parameters.

#### 5.1.3 Industrial preparation of glass

The fining of glass is a temperature‐controlled preparational process used in industry to remove unwanted bubbles.^1^, ^6^, ^38^ One form of this process involves the heating of a large vat of glass to a specific temperature at which it is held in a molten state, allowing bubbles to rise out of the melt, before being cooled again (discontinuous fining). A second form of fining process involves moving molten glass continually through a high‐temperature environment to achieve the same removal of bubbles (continuous fining). This preparation is instrumental to the production of commercial glasses, and time and energy is expended removing very small bubbles (*R* < 0.2 mm) in order to produce ‘‘flawless’’ glass.^38^

Heating programs used to remove bubbles through buoyancy effects are currently designed with the assumption of the Hadamard–Rybczynski equation^38^ (Equation 2a). The length of the isothermal hold (soak period) is altered depending on degree of refinement required (i.e., the smallest bubble needing to be removed). To remove the smallest bubbles, these soak periods could be several hours in length. Our work presented here has the potential to reduce the length of soak periods required to remove bubbles, by also accounting for bubble movements during the non‐isothermal heating and cooling ramp stages. Thus, accounting for non‐isothermal bubble rise could reduce the time and energy costs of glass refinement.

**FIGURE 9:**
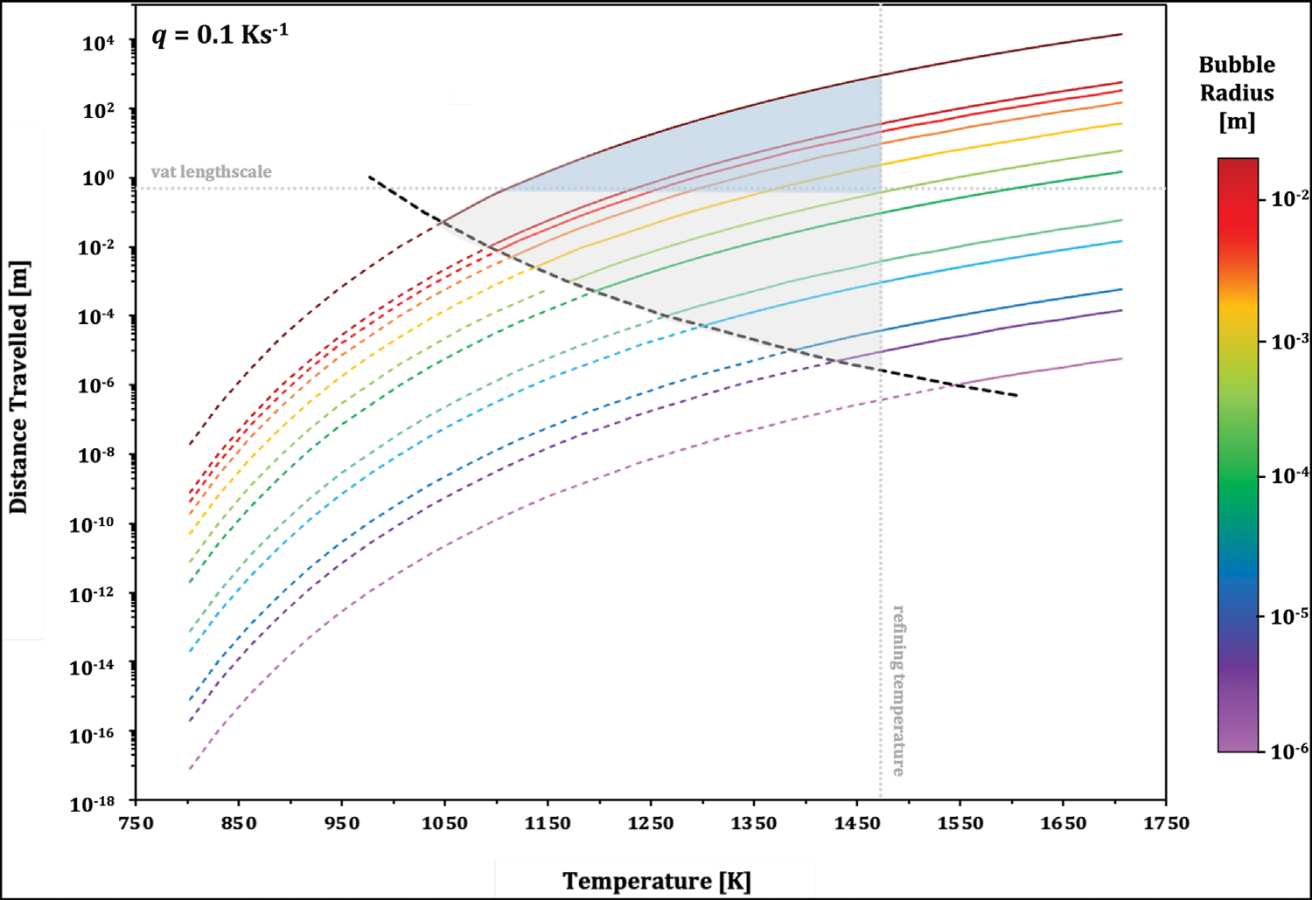
Simulations of bubble rise in Spectrum System‐96 glass during heating at 0.1 K s^−1^. The color of each line corresponds to its radius. The black dashed line shows where a bubble has risen a distance equal to its radius. Above this line, bubbles are said to have risen a significant distance during heating. Grey dashed lines show the typical temperature for glass refinement, 1427 K, and typical vertical length scale for a refining vat, 0.5 m. The grey shaded region denotes where bubble will rise a significant distance during heating, prior to any isothermal hold. The darker, blue shaded region shows where bubbles will rise a distance greater than the length scale of the refining vat during heating and will therefore be removed from the melt without need for an isothermal hold

Figure 9 shows the distance travelled by bubbles of various radii during heating at 0.1 K s^−1^ in Spectrum System‐96 glass, as simulated using our complete solution to Equation (5). Lines are added to represent a plausible isothermal hold temperature for glass refining, 1473 K (we note this is at the lower end of industrial glass refinement temperatures), and vertical length scale for a vat, 0.5 m.^1^, ^39^ This highlights that all bubbles except the smallest simulated bubble (R=1μm) move a significant distance in the time required to reach the isothermal hold temperature, where significant is defined as moving a distance equal to or greater than the bubble's radius. Bubbles experiencing significant movement before isothermal hold sit within the grey shaded region of Figure 9. For refinement processes taking place at higher temperatures (e.g., 1775 K), this region of significant movement would expand and could include even smaller bubbles.

The blue shaded region of Figure 9 takes into consideration the typical vertical length scale of a vat, thus representing the rise distance required for a bubble to be removed from the melt. All bubbles that sit within this region would therefore move free of the melt entirely during heating, and no further isothermal hold would be required. For this result for Spectrum System‐96 glass being heating at 0.1 K s^−1^ (350 K h^−1^), an isothermal hold period would only be needed if bubbles with a radius 1 mm are required to be removed. It is important to note that due to very slow rise velocities, the timescale for movement of the smallest bubbles to become significant is far longer than a plausible fining timescale, regardless of the thermal conditions applied. As a result, these smallest bubbles pose a limit to the level of refinement that can be achieved for a given glass.

**FIGURE 10:**
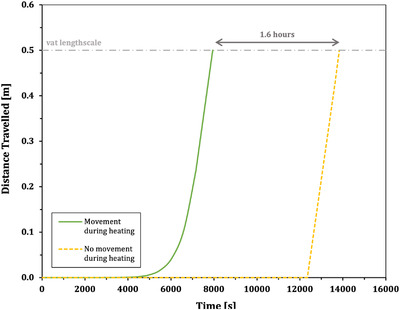
Comparison of the time required for a 1 mm radius bubble to be removed from a 0.5 m deep glass vat for an assumption that movement is negligible during heating (dashed yellow curve), and that movement occurs during heating (solid green curve). When movement during heating is accounted for, the estimated time for the bubble to be removed from the refining vat is 1.6 h shorter than when this movement is not accounted for

We envisage that this type of analysis could be used in industrial settings and adapted for different glass compositions to determine if an isothermal hold is required in the fining process. If it is found that no soak period, or a shortened soak period, is needed, this could optimize the timing of glass refining. Figure 10 demonstrates this point by showing the time for a 1 mm bubble to move the length scale of the refining vat for both our non‐isothermal solution and the isothermal Hadamard–Rybczynski solution, where bubble movement before the hold temperature is said to be negligible. The time for removal of the bubble taking into account non‐isothermal movement is 1.6 h shorter than if movement is considered to only take place during the soak period. Not only does this show the potential for significant time saving in the fining process, but could represent a potential area to reduce costs and energy use, as the vat would not need to be heated for such an extended period of time at high temperatures. Further savings would be made if bubble rise during cooling were also accounted here for using our model.

For industrial glass fining being completed on a much larger scale or at higher temperatures, it is possible that convective currents may form within the system, which could affect the distribution of heat and therefore the removal of bubbles. Whilst not considered here, it would be beneficial for future work to account for this and explore the impacts that convection may have on bubble rise and glass refinement.

### 5.2 Complex effects

Here, we deal with a simple case of spherical bubbles, where Equation (2) is valid. Larger bubbles, for which gravitational or inertial forces may be important, could lead to non‐spherical bubble with different shapes, and different functional forms for $u_{\infty }.$ ^23^ We posit that if our assumption of a changing, but spatially homogeneous temperature field is valid (i.e., Pe_T_ ≪ 1; Section 2), then our result via Equation (5) could be applied to a different $u_{\infty }$ in a different regime. However, regimes in which bubbles become non‐spherical are typically achieved for relatively large bubbles compared with the viscous and capillary regimes for spherical bubbles. As bubble size gets larger, the propensity for system changes in temperature to result in temperature gradients on the scale of the bubble increase. Therefore, there is likely to be a complex regime transition, not only to non‐spherical regimes with inertial and/or gravitational effects on $u_{\infty },\;$but also to the non‐isothermal regime in which temperature gradients occur on the bubble scale such that Pe_T_ > 1. These more complex non‐isothermal cases in which the interfacial tension, viscosity, and density may be considered a function of space around the bubble,^22^ can also result in shape deviations from spherical. Therefore, our work sits in one end‐member of a complex dynamic suite of regimes for the motion of bubbles in non‐isothermal conditions.

## 6 CONCLUDING REMARKS AND OUTLOOK

We have presented an analytical solution for non‐isothermal bubble rise which takes the form of an integrated solution of the Hadamard–Rybczynski model for $u_{\infty }$ that allows the effects of temporally changing temperature to be taken into account. We have validated the use of a fully integrated solution using an experimental methodology adapted from artistic techniques that allows bubble rise to be controlled by altering the rate of heating, cooling, or the timescale of any isothermal hold. We have also demonstrated that applying the simplest integration case, that accounts for temperature dependence of melt viscosity only, still provides a reasonable fit to observations, indicating that knowing the viscosity–temperature relationship of a glass is fundamental to modelling bubble rise in non‐isothermal conditions.

We consider some of the practical applications of the model, such as the control and design of artistic or industrial kiln‐based processes, and also some larger scaled problems such as the rise of bubbles in magmatic melts within a volcanic conduit. Whilst our model is only validated here for a laboratory scale experiment, we discuss how it might be scaled to such settings. Furthermore, this analytical integration approach could be applicable to account for other variables that may be encountered, such as special temperature change, or non‐isobaric conditions.
